# An evaluation of the completeness of safety reporting in reports of complementary and alternative medicine trials

**DOI:** 10.1186/1472-6882-11-67

**Published:** 2011-08-22

**Authors:** Lucy-Ann Turner, Kavita Singh, Chantelle Garritty, Alexander Tsertsvadze, Eric Manheimer, L Susan Wieland, James Galipeau, David Moher

**Affiliations:** 1 Ottawa Methods Centre, Clinical Epidemiology Program, Ottawa Hospital Research Institute, Ottawa Hospital, Ottawa, Ontario, Canada; 2 Centre for Integrative Medicine, University of Maryland School of Medicine, Baltimore, MD, USA

## Abstract

**Background:**

Adequate reporting of safety in publications of randomized controlled trials (RCTs) is a pre-requisite for accurate and comprehensive profile evaluation of conventional as well as complementary and alternative medicine (CAM) treatments. Clear and concise information on the definition, frequency, and severity of adverse events (AEs) is necessary for assessing the benefit-harm ratio of any intervention. The objectives of this study are to assess the quality of safety reporting in CAM RCTs; to explore the influence of different trial characteristics on the quality of safety reporting.

**Methods:**

Survey of safety reporting in RCTs published in 2009 across 15 widely used CAM interventions identified from the Cochrane Collaboration's CAM Field specialized register of trials. Primary outcome measures, the adequacy of reporting of AEs; was defined and categorized according to the *CONSORT for harms extension*; the percentage of words devoted to the reporting of safety in the entire report and in the results section.

**Results:**

Two-hundred and five trials were included in the review. Of these, 15% (31/205) reported that no harms were observed during the trial period. Of the remaining 174 trials reporting any safety information, only 21% (36/174) had adequate safety reporting.

For all trials, the median percentage of words devoted to the reporting of safety in the results section was 2.6. Moreover, 69% (n = 141) of all trials devoted a lesser or equal percentage of words to safety compared to author affiliations. Of the predictor variables used in regression analysis, multicenter trials had more words devoted to safety in the results section than single centre trials (P = 0.045).

**Conclusions:**

An evaluation of safety reporting in the reports of CAM RCTs across 15 different CAM interventions demonstrated that the reporting of harms was largely inadequate. The quality of reporting safety information in primary reports of CAM randomized trials requires improvement.

## Background

Adequate reporting of safety in primary publications of randomized controlled trials (RCTs) is a pre-requisite for accurate and comprehensive profile evaluation of conventional as well as complementary and alternative medicine (CAM) interventions. Clear and concise information on the frequency and severity of Adverse Events (AEs) is necessary in assessing the benefit-harm ratio when administering any intervention. In 2007, it was estimated that 38.3% of American adults use some form of CAM [1]. With such high usage rates, it is essential that the evidence is of the highest quality and that safety information is reported in sufficient detail.

In 1998 and 2001, Ioannidis and Lau published a study evaluating the reporting of safety data in 7 medical areas [2,3]. This evaluation concluded that the reporting of safety data was largely inadequate. Moreover, the absolute space given to author affiliations was often more than that given to the reporting of safety. Ioannidis called for the replication of such an evaluation across many fields.

While there have been recent advances in conventional medicine to standardize the collection, analysis and reporting of efficacy data in clinical trials [4], and more recently, the reporting of safety [5], there is little evidence regarding adequacy of reporting within CAM trials. Referencing Ioannidis' objectives and methods, this study evaluates safety information across 15 CAM interventions. Our aim was to assess the adequacy of safety reporting in CAM trials, to gain a better understanding of the predictors of adequate reporting, and to extend and assess adherence of CAM RCTs to the CONSORT recommendations for harms extension [6].

## Methods

### Trial Database

We searched the Cochrane Complementary Medicine Field (CAM Field) Specialized Register of trials [7] and obtained citations to all CAM RCTs published in 2009 pertaining to 15 CAM intervention categories. The inclusion of study reports was not restricted by study sample size or language of publication. All non-full text reports were excluded along with reports which could be categorized by more than one intervention; had AEs as a primary outcome; or for which full text articles were not locally available.

### Selecting Interventions

The top 5 most commonly used CAM therapies according to the National Health Interview Survey 2007 were the following: 1. Natural products (17.7%); 2. deep breathing (12.7%); 3. meditation (9.4%); 4. Chiropractic and osteopathic (8.6%); and 5. massage (8.3%)[8]. Because natural products are by far the most commonly used CAM therapy, we focused on that CAM topic area, however only included the top 10 most commonly used natural products in our sample [9]. There is wide acceptance of minimal, if any, AEs associated with deep breathing and meditation; hence these topics were excluded from our study. In addition, we decided to include acupuncture and Chinese herbal medicine CAM topic areas despite not being among the top 10 most commonly used CAM therapies, as both topics are of popular interest, widely used globally [10,11] and there is much available literature [12].

As such, 15 CAM interventions classified according to categories developed by the Cochrane CAM field, were chosen based on their respective impact, accessibility and frequency of use [13]. Interventions include: acupuncture, massage, chiropractry, traditional Chinese herbal medicine and other traditional medicine, fish oil/omega 3, glucosamine, flaxseed, ginseng, combination herbal products, ginkgo biloba, garlic supplements, coenzyme Q-10, echinacea and chondroitin. Acupuncture was defined as a needling intervention, breaking the skin [14], as such Moxibustion, TENS (transcutaneous electrical nerve stimulation), laser acupuncture and acupressure trials were not included.

### Study selection and data extraction

Retrieved trials were screened and data extracted using online review software, DistillerSR. Title and abstract screening was conducted in duplicate by two of three review authors and subsequent full text articles were retrieved and screened independently by two of five reviewers, any disagreements were discussed and remaining conflicts were resolved by a third independent author.

Standardized data extraction forms were created to ensure all reported data was collected from the trials meeting inclusion criteria. All data to be collected was determined a priori. Data extraction was completed independently and verified by two authors by taking a 10% random sample of trials. A discrepancy of 10% in the number of words extracted was defined to be acceptable, however only two trials exceeded this cut off. Only 6.5% of all extracted data was modified. There were no amendments to outcomes which were considered to threaten the reliability of the data and as a result, no further explicit verification by study was conducted.

### Evaluation and Analysis

#### Descriptive Characteristics

Descriptive characteristics of included trials were based on variables which could be potentially used to predict qualitative and quantitative measures of reporting safety data. Founded upon the variables considered by Ioannidis with additions and modifications subject to the context of this evaluation, descriptive characteristics included: Total sample size; whether or not a trial was reported as double-blind; whether or not the trial reported significant results for efficacy; type of funding, industry versus non-industry; longest duration to follow up subject to CAM trials (a 6 month divide was deemed appropriate). We recorded information to assess if more words or more adequate reporting was given to safety information in paediatric trials, therapeutic trials (versus preventative) studies which were in combination with conventional medicine (i.e. Acupuncture and Chemotherapy) and studies which were conducted at multiple centres. We also collected 2009 impact factor information for publishing journals of included studies; this information was collected from the journal websites.

### Adequacy of Safety Reporting: Qualitative and Quantitative Measures

**Figure 1:**
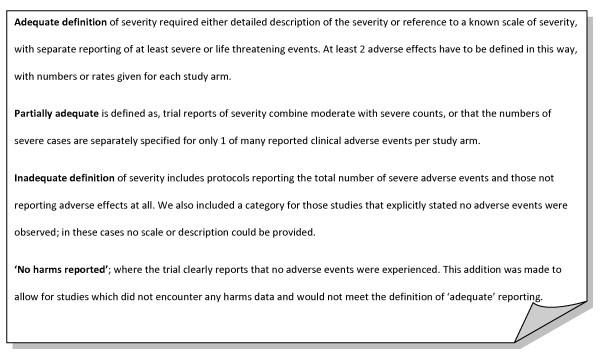
**Defining qualitative parameters for adequacy of reporting**.

Qualitative and quantitative components of AEs reporting offer complementary information. The measures developed by Ioannidis in 2001 were used as guidance in the selection of qualitative and quantitative measures for this evaluation. We measured the following two qualitative components: 1. the reporting of dropouts due to AEs and whether the total number of dropouts were reported; and 2. Whether severity of the described AEs were reported, such reporting was classified as adequate, partially adequate, inadequate or no harms reported (Figure 1).

In order to determine the relative emphasis of safety reporting in these CAM reports we assessed quantitative measures, namely, the percentage of words reporting safety in the body of the text and the percentage of words reporting safety in the results section. The full body of the text was defined as all words in the trial report excluding the title, abstract, acknowledgements, appendices and affiliations. Sentences including any words reporting harms were not broken. Like Ioannidis, we compared the relative reporting of safety in the body of the text with the text given to author affiliations. We also extracted the number of words devoted to the reporting of safety and the total number of words in the results section to compute the percentage of words devoted to safety in the results section.

### CONSORT for Harms

**Figure 2:**
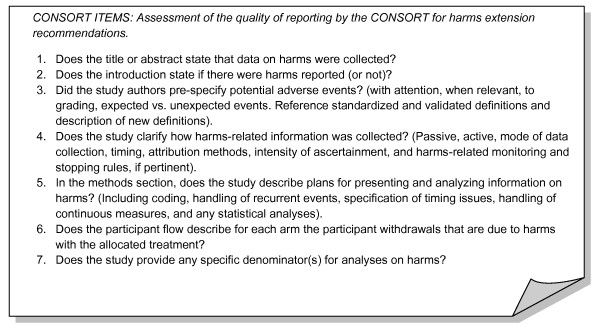
**CONSORT for Harms Data Recommendations**.

In 1996, a group of international experts published the CONSORT Statement [15]. This reporting guideline was updated in 2001 and more recently in 2010 [16,17]. Several extensions to facilitate reporting of other trial designs, such as cluster trials, and other types of data including harms, have been developed. We applied the *CONSORT for harms extension *collecting data on each of the first seven recommendations described as part of this evaluation (Figure 2).

### Data Analysis

All descriptive characteristics are reported by CAM area as the frequencies and percentages of trials with the exception of sample size and 2009 journal impact factor for which median and inter-quartile ranges are reported. Descriptive characteristics were used as predictor variables in both least-squares multiple regressions and binary logistic regressions. The qualitative and quantitative measures of percentage of words devoted to the reporting of safety in the results section, and adequacy of reporting were respective dependent variables. Multiple least-squares analyses for each predictor were run adjusting for CAM area by dummy variables. Logistic regressions of clinical AEs (no harms reported and adequate reporting versus partially adequate and inadequate) were also run. Similarly, all analysis was adjusted for CAM therapy using dummy variables. All analysis was conducted using Minitab^® ^Version 16.1.1.

## Results

### Search results and included trials

**Figure 3:**
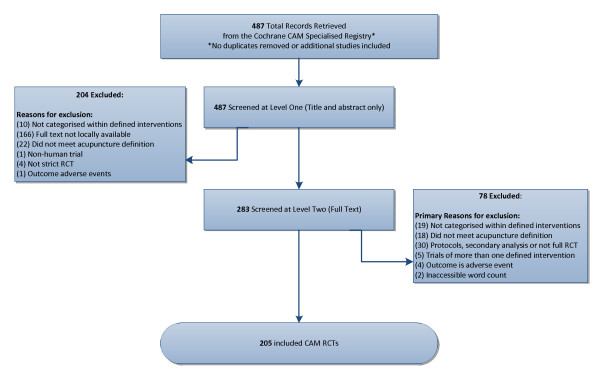
**Study Flow Diagram**.

A total of 487 trials were identified. Of these, 166 trials were excluded as full text articles were not available; 29 trials were excluded as they did not fall under any of the 15 pre-defined CAM intervention categories; 87 more trials were excluded for not meeting our eligibility criteria (e.g., protocols, non-randomized trials, secondary publications, primary outcome AEs, incalculable word count, non-human study). Two of the fifteen CAM areas, echinacea and chondrotin, did not yield any trials. The remaining 205 trials, representing 13 different CAM areas, were included in the review (Figure 3).

### Descriptive Characteristics of Included Trials

Descriptive characteristics of included trials are presented in Table 1. Of the 205 included trial reports, 48% (n = 99) were described as double-blind. The majority of trials (74%, n = 152) evaluated a therapeutic CAM intervention versus a preventative intervention, and 23% (n = 47) of interventions were a combination of CAM and non-CAM treatments. Only five of the 13 CAM categories (acupuncture, Chinese herbal medicine, co-enzyme Q-10, combination herbal products and fish oil) included a paediatric population.

**Table 1:** Characteristics of included CAM RCTs

Intervention	Sample Size Median (Q1-Q3), Total	Double-blind No. (%)	Significant results for efficacy No. (%)	Industry funded No. (%)	Longest duration to follow up ≥ 6 m, No. (%)	Paediatric population No. (%)	Therapeutic studies No. (%)	Combined Studies No. (%)	Multicenter trials No. (%)	Journal IF > 5, No. (%)
**Acupuncture, n = 75**	60 (32-102) 8,717	16 (21)	58 (77)	5 (7)	9 (12)	4 (5)	71 (95)	23 (31)	11 (15)	9 (12)

**Chinese herbal Medicine, n = 46**	71 (48-98) 6,170	20 (43)	36 (78)	3 (6.5)	6 (13)	2 (4)	32 (70)	12 (26)	11 (24)	1 (2)

**Chiropractic, n = 6**	32 (28-47) 402	1 (17)	4 (67)	1 (17)	1 (17)	-	5 (83)	2 (33)	1 (17)	-

**Coenzyme Q-10, n = 8**	56 (34-166) 789	8 (100)	6 (75)	2 (25)	4 (50)	2 (25)	5 (63)	-	1 (13)	2 (25)

**Combination herbal products, n = 11**	85 (52-97) 882	9 (82)	6 (55)	6 (55)	2 (18)	1 (9)	9 (82)	1 (9)	3 (27)	-

**Fish oil/Omega 3, n = 29**	64 (33-105) 2,671	25 (86)	14 (48)	7 (24)	10 (34)	4 (14)	14 (48)	6 (21)	3 (10)	2 (7)

**Flax Seed, n = 6**	48 (38-68) 321	4 (67)	4 (67)	1 (17)	1 (17)	-	2 (33)	-	-	-

**Garlic supplements, n = 2**	542 (303-780) 1,083	2 (100)	2 (100)	1 (50)	2 (100)	-	-	1 (50)	-	-

**Gingko Biloba, n = 4**	80 (56-835) 3,243	2 (50)	2 (50)	1 (25)	1 (25)	-	3 (75)	-	1 (25)	1 (25)

**Ginseng, n = 6**	62 (30-85) 394	5 (83)	2 (33)	3 (50)	1 (17)	-	2 (33)	1 (17)	-	2 (33)

**Glucosamine, n = 3**	22 (19-122) 260	1 (33)	2 (67)	-	2 (67)	-	3 (100)	-	1 (33)	-

**Massage, n = 1**	- 35	1 (100)	1 (100)	-	-	-	-	-	-	-

**Traditional medicine, n = 8**	66 (48-108) 643	5 (63)	7 (88)	2 (25)	-	-	6 (75)	1 (13)	3 (38)	-

TOTAL, (n = 205)	63 (36-100) 25,610	99 (48)	144 (70)	33 (16)	39 (19)	13 (6)	152 (74)	47 (23)	35 (17)	17 (8)

'-' Denotes zero trials

'NR' Not reported

Seventy six (37%) trials did not report a source of funding. Thirty three (16%) trials were funded by industry, 47 (23%) by government, 49 (24%) by academic institutions. Eighty three (40%) trials did not report the longest duration of follow up. Only 35 (n = 17%) of trials were multi-centered. The median 2009 journal impact factor for all publications was 1.86 (range 0.42 to 9.81). One study, published in JAMA, was not included in the calculation due to a comparatively large 2009 impact factor.

### Adequacy of safety reporting

#### Qualitative measures

Fifteen percent (31/205) of trials explicitly reported that no AEs had been experienced during the trial. More than half of all trials (56%, 114/205) had inadequate reporting of safety data. Of these, 69% (79/114) had no words dedicated to the reporting of harms anywhere in the trial report. In 18% (36/205) of all trials the reporting of safety was deemed to be adequate and in 12% (24/205) of the trials, the safety reporting was considered as partially adequate. Although treatment discontinuation was reported in 79% (162/205) of trials, the number reporting details on dropouts due to AEs was less at 60% (124/205) (Table 2).

**Table 2:** Safety Reporting: Qualitative measures

	Clinical Adverse Events, No. (%)				Discontinuations		
**Intervention**	**Adequate**	**Partially adequate**	**Inadequate**	**No harms reported**	**Total reported**	**Due to adverse events**	**Reporting adverse events when reporting discontinuations**

**Acupuncture, n = 74**	8 (11)	12 (16)	46 (61)	9 (12)	57 (76)	45 (60)	45/57

**Chinese herbal** **Medicine, n = 46**	10 (22)	5 (11)	23 (50)	8 (17)	32 (70)	31 (67)	31/32

**Chiropractic, n = 6**	2 (33)	1 (17)	2 (33)	1 (17)	5 (83)	5 (83)	5/5

**Coenzyme Q-10, n = 8**	1 (13)	-	4 (50)	3 (38)	7 (88)	4 (50)	4/7

**Combination herbal products, n = 11**	3 (27)	-	6 (55)	2 (18)	9 (82)	8 (73)	8/9

**Fish oil/Omega 3, n = 29**	3 (10)	3 (10)	19 (66)	4 (14)	27 (93)	15 (52)	15/27

**Flax Seed, n = 6**	2 (33)	-	4 (67)	-	4 (67)	3 (50)	3/4

**Garlic supplements, n = 2**	-	-	2 (100)	-	2 (100)	-	0/2

**Gingko Biloba, n = 4**	2 (50)	-	-	2 (50)	4 (100)	3 (75)	3/4

**Ginseng, n = 6**	2 (33)	-	4 (67)	-	6 (100)	5 (83)	5/6

**Glucosamine, n = 3**	-	-	2 (67)	1 (33)	2 (67)	1 (33)	1/2

**Massage, n = 1**	-	-	1 (100)	-	-	-	-

**Traditional medicine, n = 8**	3 (38)	3 (38)	1 (13)	1 (13)	7 (88)	4 (50)	4/7

Total (n = 205)	36 (18)	24 (12)	114 (56)	31 (15)	162 (79)	124 (60)	124/162

#### Quantitative measures

The median percentage of words for reporting safety in the full body of the text was 0.94 (Q1-Q3 0.00-3.26). The median percentage of words in the results section devoted to the reporting of safety was 2.55 (Q1-Q3 0.00-11.07). Less than 30% (n = 60) of trials devoted an equal or greater percentage of words for the reporting of safety than to author affiliations (Table 3). In 141 (69%) trials more words were given to the reporting of author affiliation information than safety information, in 4 (2%) trials the percentage of words were considered to be the same (within 0.1%), and 60 (29%) trials gave more space to the reporting of harms relative to the reporting of author information.

**Table 3:** Safety of Reporting: Quantitative measures

	Percentage of words for safety reporting		Relative emphasis on safety reporting, No. (%)			Other sources of safety data, No. (%)		
**Intervention**	**Body of the text, median (Q1-Q3)**	**Results section, median (Q1-Q3)**	**Safety < affiliations**	**Safety = affiliations**	**Safety > affiliations**	**≥ 1 Table for safety data**	**≥ 1 Figure for safety data**	**Additional resources**

Acupuncture, n = 75	0.7 (0.0-2.0)	1.2 (0.0-9.0)	56 (75)	1 (1)	18 (24)	7 (9)	3 (4)	2 (3)

Chinese herbal Medicine, n = 46	1.5 (0.0-5.1)	7.1 (0.0-15.7)	27 (59)	1 (2)	18 (39)	6 (13)	-	-

Chiropractic, n = 6	2.0 (0.1-3.7)	1.8 (0.0-15.3)	3 (50)	-	3 (50)	1 (17)	1 (17)	-

Coenzyme Q-10, n = 8	1.2 (0.0-3.1)	1.9 (0.0-5.1)	6 (75)	-	2 (25)	1 (13)	1 (13)	-

Combination herbal products, n = 11	2.7 (1.0-4.5)	6.6 (0.5-13.3)	5 (45)	1 (9)	5 (45)	2 (18)	2 (18)	1 (9)

Fish oil/Omega 3, n = 29	0.0 (0.0-1.8)	0.0 (0.0-6.9)	21 (72)	1 (3)	7 (24)	1 (3)	3 (10)	1 (3)

Flax Seed, n = 6	0.0 (0.0-1.4)	0.0 (0.0-6.0)	5 (83)	-	1 (17)	-	-	-

Garlic supplements, n = 2	0.0 (0.0-0.0)	0.0 (0.0-0.0)	2 (100)	-	-	-	-	2 (100)

Gingko Biloba, n = 4	1.6 (1.5-1.9)	9.0 (7.4-11.7)	4 (100)	-	-	-	-	1 (25)

Ginseng, n = 6	0.5 (0.0-2.1)	2.0 (0.0-0.6)	5 (83)	-	1 (17)	1 (17)	-	-

Glucosamine, n = 3	0.0 (0.0-2.4)	0.0 (0.0-3.5)	2 (67)	-	1 (33)	-	-	-

Massage, n = 1	-	-	1 (100)	-	-	-	-	-

Traditional medicine, n = 8	3.6 (1.1-8.4)	9.1 (3.3-21.6)	4 (50)	-	4 (50)	2 (25)	-	-

TOTAL (n = 205)	**0.9 (0.0-3.3)**	**2.6 (0.0-11.1)**	**141 (69)**	**4 (2)**	**60 (29)**	**21 (10)**	**10 (5)**	**7 (34)**

For all trials the percentage of words for reporting author affiliations was statistically greater than that for reporting safety data (Wilcoxon test, p < 0.001). Two of the thirteen CAM areas, Acupuncture and Fish Oil/Omega 3 (P < 0.001 and P < 0.01 respectively) also showed significant differences in the number of words devoted to safety and affiliations, with more words given to the reporting of author affiliations. Only 10% of trials reported safety data in at least one table, and 5% of all trials reported safety data in a figure. Seven (3%) trials referred to additional external resources (online or archived) with additional safety data.

### CONSORT for Harms

In general, we found a low compliance with seven CONSORT for harms recommendations. Of these recommendations, 'participant flow' yielded the highest frequency for reporting in 30% (n = 61) of trials. The lowest compliance (< 5% of all trials) was observed with recommendation 2, the reporting harms information in the introduction (Table 4).

**Table 4:** Reporting of Safety in accordance with CONSORT for Harms Recommendations

	CONSORT for Harms Recommendation, No. (%)						
**Intervention**	**1**	**2**	**3**	**4**	**5**	**6**	**7**

**Acupuncture, n = 74**	13 (17)	2 (3)	5 (7)	9 (12)	6 (8)	29 (39)	16 (21)

**Chinese herbal Medicine, n = 46**	16 (35)	3 (7)	7 (15)	11 (24)	5 (11)	9 (20)	10 (22)

**Chiropractic, n = 6**	2 (33)	1 (17)	-	-	1 (17)	2 (33)	2 (33)

**Coenzyme Q-10, n = 8**	1 (13)	-	1 (13)	1 (13)	-	3 (38)	1 (13)

**Combination herbal products, n = 11**	5 (45)	1 (9)	-	2 (18)	-	4 (36)	2 (18)

**Fish oil/Omega 3, n = 29**	1 (3)	1 (3)	1 (3)	4 (14)	-	8 (28)	2 (7)

**Gingko Biloba, n = 4**	-	-	-	1 (25)	-	1 (50)	-

**Ginseng, n = 6**	1 (17)	-	-	1 (17)	-	2 (33)	2 (33)

**Glucosamine, n = 3**	1 (33)	-	-	1(33)	-	-	-

**Traditional medicine, n = 8**	4 (50)	1 (13)	-	3 (38)	-	1 (13)	2 (25)

TOTAL (n = 205)	43 (21)	9 (4)	13 (6)	34 (17)	12 (6)	61 (30)	37 (18)

Please Note: None of the trials of flax seed, garlic supplements or massage adhered to any of the CONSORT recommendations

### Regression Analysis

In univariate analysis, the percentage of words devoted to the reporting of safety in the results section increased significantly for multicenter trials (p = 0.045), (Table 5). In multivariable analysis, multicenter trials were retained as a statistically significant predictor for increasing the percentage of words in the results section of a trial irrespective of CAM area. It is of interest that Coenzyme Q-10 (p = 0.017), Fish oil/Omega 3 (p = 0.018) and Flax Seed (p = 0.043) all saw significant reductions in the percentage of words in the results section reporting safety. Logistic regressions did not yield any significant increases in odds of adequate reporting of safety based on predefined predictor variables. Although non-significant, industry funded trials odds of adequate reporting of harms were 2.49 times (95% CI, 0.98 to 6.32) that of non-industry funded trials; and multicenter trials had an odds of 1.89 (95% CI, 0.98 to 1.43) times those of single centre trials of adequately reporting safety information.

**Table 5:** Regression Analysis

	Increase in percentage of words devoted to the reporting of safety in the results section^Ŧ^	Adequacy of reporting
**Predictors**	**Least-square regressions (Adjusted^¥^) Effect Size (95% CI)**	**Logistic regressions (Adjusted) OR (95% CI)**

**Sample Size (per 10 unit increase)**	0.02 (-0.04-0.07)	1.00 (1.00-1.00)

**Double Blind**	2.86 (-1.04-6.76)	1.05 (0.47-2.31)

**Significant results for efficacy**	-1.93 (-5.36-1.49)	1.18 (0.03-1.40)

**Industry Funded**	0.27 (-4.04-4.58)	2.49 (0.98-6.32)

**Longest duration to follow-up, ≥ 6 m**	-0.07 (-4.04-3.91)	0.99 (0.42-2.33)

**Paediatric population**	-4.50 (-11.07-2.07)	0.56 (0.11-2.90)

**Therapeutic studies**	3.21 (-0.84-7.26)	1.81 (0.73-4.48)

**Combined studies**	-0.32 (-4.03-3.39)	0.79 (0.34-1.81)

**Multicentre**	4.02 (0.12-7.92)*	1.89 (0.84-4.27)

**2009 Journal Impact Factor (per 10 unit increase)**	1.05 (-6.01-8.18)	1.19 (0.98-1.43)

*Statistically significant, p = 0.045, ^¥^All regressions adjusted for CAM intervention, ^Ŧ^Multiple least-squares regressions for each predictor detailed. Adjusted forced entry (p < 0.1) and stepwise elimination and entry models retain multicenter (p = 0.03) and therapeutic studies (0.02).

## Discussion

In general, findings of this evaluation indicate that the safety reporting across trials of CAM interventions is inadequate and often ignored altogether. Largely, the percentage of words devoted to the reporting of safety was equal to or less than that devoted to author affiliations. Adequate reporting of AEs was only observed in 18% of trials, with a further 9% of trials reporting that no harms were observed. Thirty-nine percent of trials gave no mention whatsoever to potential AEs. The regression models showed that the percentage of words devoted to the reporting of safety was significantly greater for the reports of multicenter trials compared to the reports of single centered trials; but otherwise none of the considered predictor variables were significant in modelling the percentage of words in the results section or the adequacy of reporting.

Consistent with the somewhat acceptable reporting of dropouts due to AEs, the assessment of adherence to the *CONSORT for harms extension *demonstrates that participant flow diagrams are the most successfully reported recommendation; however, this is under 30% of trials. Notably, perhaps, only just over 20% of trials discuss AEs in the abstract (Recommendation 1). Similar to the adequacy of reporting of safety outcome, the compliance of trials to recommendations of the CONSORT extension is weak.

These main findings are somewhat consistent with Ioannidis' evaluation of seven medical areas from a decade ago. The percentage of trials adequately reporting safety information in CAM trials was lower (18% of the total, or 21% of those trials not explicitly reporting no harms, versus 39%). Our finding that a larger percentage of words were devoted to author affiliations compared to AEs mirrors Ioannidis' finding that more publication area is devoted to author affiliations than to AEs. The overall conclusions of both evaluations are consistent in that the percentage of trials reporting harms and the adequacy by which they are reported is largely inadequate irrespective of defined predictors.

Inadequate reporting of harms also has consequences for systematic reviewers. The synthesis of harms from individual trials will be seriously compromised by inadequate reporting. The net effect is to negate the altruistic volunteerism of trial participants; there are important ethical and moral reasons to improve the quality of reporting of all research [18].

We would like to note some limitations to our evaluation. This is a sample across both physical interventions and natural products, therefore we may be assessing interventions for which AEs rarely occur [19,20] (or rarely in comparison to drug interventions), or by which symptoms of overdose or usage are very minor and would not require medical attention. As such authors may not have considered reporting safety information. This by no means justifies the lack of adequacy of reporting of safety for these trials. If it is deemed by the trialist that no such AEs could occur independently or by interaction with other medications [21,22], this should be sufficiently described in the trial report.

Due to limited resources, only locally available trials were included. This excluded a large number of trials reported in languages other than English. However, based on the similarity of included results of the reporting of adequacy across all CAM areas, it is unlikely that the exclusion of these trials would greatly affect our results. We have drawn comparison between proportion of words given to author affiliation and safety of reporting. Our results suggested that 69% of trials devoted more words to author affiliations than reporting of safety, and 29% devoted more words to safety than affiliation. This discrepancy may be somewhat confounded by a number of external influences such as editorial policy, number of investigators involved with a study and for the trials which report no harms. We feel however, that although the difference may not be as large as reported, this does not negate the finding that more trials devoted more space to author affiliations than to the reporting of safety.

We would also like to note that not all trials were included in the regression analysis (n = 172). Trials with missing data on duration to follow up and trials published in new journals without 2009 impact factors were omitted. The fit of all regression models was poor. This would suggest that either there are other variables associated with the quality of reporting of safety data which we did not consider, or these characteristics can by no means predict the quality of reporting, and that ultimately, the reporting of AEs is independently subject to the study author.

It could be hypothesised that there would be an association between journal endorsement of the CONSORT statement and trial adherence to the CONSORT for harms extension. Hopewell et al. report in 2008 the adherence to the CONSORT for harms statement is mentioned in only 3 of 165 high impact medical journals [23]. It is likely that this lack of endorsement is consistent within our sample; we did not investigate this association in this evaluation as the proportion of adhering journals would be sparse.

This study provides strong evidence, including many trials across many well established CAM interventions, that the reporting of safety data is inadequate. While there have been ongoing strides in conventional medicine to standardizing the collection, analysis and reporting of efficacy data in clinical trials [4], and more recently, the reporting of safety [5]; the Cochrane CAM Field has found no other evidence regarding the adequacy of such reporting within CAM trials. Hence, this study is the likely the first to provide such information.

Given the increasing use of CAM, it is necessary to improve such reporting to enable accurate and objective marketing and use of such treatments or products. Although large robust trials have a lot of information to report, it is not acceptable for the reporting of safety to be overlooked. Sufficient guidance must be provided to authors to ensure that the safety data is comprehensive and accessible to readers even when there have been no observed AEs.

The *CONSORT for harms extension *is a valuable resource for improving the quality of reporting of harms in conventional medicine trials. However, it is clear that there is a need for the use of such guidance to become standard procedure for authors and editors when presenting findings for all trials. Few journals explicitly endorse CONSORT for harms or other reporting guidelines. Parenthetically, there is growing evidence that use of reporting guidelines is associated with improved quality of reporting [24-26]. Plint and colleagues reported a systematic review of eight trials evaluating the use of the CONSORT checklist and found that its use was associated with improved quality of reporting. A recent update of this review [27] now includes 49 studies examining almost 9000 trials provides strong stronger support for the association between use of the CONSORT checklist and improved reporting.

## Conclusions

An evaluation of safety reporting in the reports of CAM RCTs across 15 different CAM interventions demonstrated that the reporting of harms was largely inadequate. The quality of reporting safety information in primary reports of CAM randomized trials requires improvement. We hope that these data will impress journal editors who, in turn will now endorse reporting guidelines as an important way to improve the quality of reporting harms.

## Competing interests

The authors declare that they have no competing interests.

## Authors' contributions

All authors contributed to the concept and design of the study. AT participated in the conception, design, and interpretation of findings and drafting of the manuscript. CG participated in conception, data extraction, interpretation of findings and manuscript drafting. DM conceived of the study, participated in design, coordination, and reviewed the manuscript. EM participated in conception, provided CAM expertise and resources, and reviewed the manuscript. JG participated in conception, screening of articles, data abstraction, and reviewed the manuscript. KS participated in design and conception, screening, data extraction, summarising and interpreting findings and manuscript drafting. LT coordinated the project, was involved with conception and design, screening, data extraction, statistical analysis, interpretation of findings and drafted the manuscript. LSW participated in conception, provided CAM expertise and resources, and reviewed the manuscript. All authors read and approved the final manuscript.

## Pre-publication history

The pre-publication history for this paper can be accessed here:

http://www.biomedcentral.com/1472-6882/11/67/prepub
